# Frequency and levels of regulated and emerging mycotoxins in silage in Poland

**DOI:** 10.1007/s12550-018-0327-0

**Published:** 2018-08-22

**Authors:** L. Panasiuk, P. Jedziniak, K. Pietruszka, M. Piatkowska, L. Bocian

**Affiliations:** 1 Department of Pharmacology and Toxicology, National Veterinary Research Institute, Partyzantow Avenue 57, 24-100 Pulawy, Poland; 2 Center for Analytical Chemistry, Department of Agrobiotechnology (IFA-Tulln), University of Natural Resources and Life Sciences, Vienna, Konrad Lorenz Str. 20, 3430 Tulln, Austria; 3 Department of Epidemiology and Risk Assessment, National Veterinary Research Institute, Partyzantow Avenue 57, 24-100 Pulawy, Poland

**Keywords:** Silage, Mycotoxins, Co-occurrence, LC-MS/MS, Correlation

## Abstract

In this study, 120 silage samples collected in 2015 from farms in Poland were analysed by a multimycotoxin method based on liquid chromatography coupled with tandem mass spectrometry. The study included toxins which are regulated within the European Union (aflatoxins, deoxynivalenol, fumonisins, T-2/HT-2 toxins, ochratoxin A and zearalenone) and non-regulated mycotoxins (enniatins, beauvericin, 8-ketotrichothecenes, sterigmatocystin, zearalenone derivatives). All silage samples were positive for at least one mycotoxin, and 61% of samples contained five or more mycotoxins simultaneously. The most frequently detected toxins were deoxynivalenol, nivalenol, zearalenone, enniatins and beauvericin, although the levels of these toxins were relatively low. The mean concentration of deoxynivalenol and zearalenon was 406 and 80.6 μg/kg, respectively, and two toxins were positive-correlated. This is the first study that provides information about emerging mycotoxins contaminating silage in Poland.

**Electronic supplementary material:**

The online version of this article (10.1007/s12550-018-0327-0) contains supplementary material, which is available to authorized users.

## Introduction

Silage is one of the most common feedstuff for ruminants in Europe. In 2015, the area of land harvested in Poland for this purpose exceeded 500.000 ha, and is still increasing each year (Central Statistical Office of Poland 2016). Silage is mostly composed of maize, grass, clover, sugar beet tops, alfalfa and milo (Storm et al. 2008). The ensiling process allows for preservation of fodder for livestock for longer periods of time, without degradation, and with minimum loss of nutrients (Tangni et al. 2013). It enables its use as forage during the periods of feed scarcity (Alonso et al. 2013).

However, silage can become contaminated with toxigenic fungi, either pre-harvest (e.g. *Alternaria* spp. and *Fusarium* spp.), post-harvest (e.g. *Penicillium* spp.) (Rasmussen et al. 2010) or at both times (e.g. *Aspergillus* spp*.*). The occurrence of these fungal contaminants depends on many factors, such as climate, storage conditions and agricultural practice (Storm et al. 2008). Under specific conditions, growth of toxigenic moulds can result in the production of mycotoxins. The intake of these secondary metabolites can exert several adverse effects on livestock animals (Scudamore and Livesey 1998). Therefore, the occurrence of mycotoxins in livestock animals is one of the most serious health threats in agriculture. Moreover, production of feedstuff without any mycotoxins is very difficult (Wambacq et al. 2016).

Hundreds of mycotoxins are known of (Berthiller et al. 2007), but European Union regulation on feed has so far been established only for aflatoxins (AFB_1_, AFB_2_, AFG_1_, AFG_2_) by Directive 32/2002 (European Communities 2002), additional “guidance values” have been published by the European Commission for several other compounds, namely deoxynivalenol (DON), fumonisins (FB_1_, FB_2_), ochratoxin A (OTA), zearalenone (ZEN) (European Commission 2006) and for T-2 and HT-2 toxins (European Commission 2013). Because there are no specific regulations on mycotoxins in silage (e.g. grass silage, only for maize-based product guidance value is available), currently recommended levels for animal feed could also be considered as guidelines for silage (Cheli et al. 2013). Regarding to DON and ZEN is recommended not to exceed 12 mg/kg and 3 mg/kg, respectively.

In recent years, researchers have additionally paid more attention to the presence of “emerging mycotoxins” in food and feed, especially for the enniatins (ENNs) and beauvericin (BEA). Data on the toxicity and occurrence of emerging mycotoxins are limited, and further investigation of these compounds is needed for a proper risk assessment. Nevertheless, there have been some studies describing their potential implications for food safety (EFSA 2014a). Based on recent scientific opinion of European Food Safety Authority (EFSA), some “opinion” papers about the risk to human and animal health to the presence of regulated, modified and emerging mycotoxins have been published (EFSA 2011, 2013a, b, 2014b, 2017a, b).

The determination of mycotoxins in silage is also an analytical challenge. Silage has a complex matrix that contains many compounds, such as organic acids, sugars, chlorophyll and others, that are difficult to remove using sample extract preparation (Rasmussen et al. 2010). Hence, it is necessary to develop a suitable method of analysis for mycotoxins in silage. Altogether, several multi-analyte methods for the simultaneous determination of mycotoxins in silage do exist, and have been described in the literature so far, mostly based on liquid chromatography with tandem mass spectrometry (LC-MS/MS). This core technique can provide the highest sensitivity and specificity, enabling detection of low levels of mycotoxins in various samples and reducing the number of sample preparation steps and analysis time (Wang et al. 2016).

Until now, most researchers paid attention on mycotoxins occurrence in grains, cereals (Monbailu et al. 2010; Schenzel et al. 2012; Kovalsky et al. 2016; Abdallah et al. 2017) and maize silage (Dagnac et al. 2016; Gallo et al. 2016; Grajewski et al. 2012; Kosicki et al. 2016; Storm et al. 2014; Zachariasova et al. 2014). Only few studies on the occurrence of mycotoxins in grass silage have been published (Driehuis et al. 2008; McElhinney et al. 2016). In the aforementioned studies, the research was focused mainly on regulated mycotoxins, with *Fusarium* toxins the most frequently detected compounds. Data on emerging toxins are scarce, and further surveys are needed for a proper risk assessment. Therefore, the aim of this study was to assess the contamination levels of silage in Poland, and to study possible correlations between different toxins.

## Materials and methods

### Chemicals and reagents

Acetonitrile (ACN, analytical grade), methanol (MeOH, LC-MS grade), acetic acid and C18 bulk sorbent were purchased from J.T. Baker of Avantor Performance Materials (Netherlands). Formic acid and ammonium acetate (LC/MS grade) were supplied by Sigma-Aldrich (Germany). Magnesium sulphate was obtained from Chempur (Poland). Water was purified by a Milli-Q apparatus (USA).

### Standard solutions

From Sigma-Aldrich (Germany), the standards were obtained for AFB_1_, AFB_2_, AFG_1_, AFG_2_, 3-acetyldeoxynivalenol (3-AcDON), 15-acetyldeoxynivalenol (15-AcDON), citrinin (CIT), beauvericin (BEA), diacetoxyscirpenol (DAS), DON, enniatins A (ENN A), A_1_ (ENN A_1_), B (ENN B), B_1_ (ENN B_1_), FB_1_ and FB_2_, fusarenon X (FUS-X), nivalenol (NIV), OTA, sterigmatocystin (STC), HT-2, T-2, ZEN and β-zearalenol (β-ZEL). All standards were stored according to their manufacturer’s recommendations. Primary standard stock solutions were prepared: in acetonitrile for 3-AcDON, 15-AcDON, DON, FUS-X, HT-2, STC, T-2 and ZEN; in methanol for AFs, ENNs, NIV, OTA and β-ZEL and in 50% solution of ACN in H_2_O for FB_1_ and FB_2_. The stock solutions were used to prepare working standard solutions containing the 24 analytes in concentrations corresponding to the lowest regulatory levels or guidance levels (GL) in feedstuffs (Supplementary Table S2).

### Samples

**Fig. 1:**
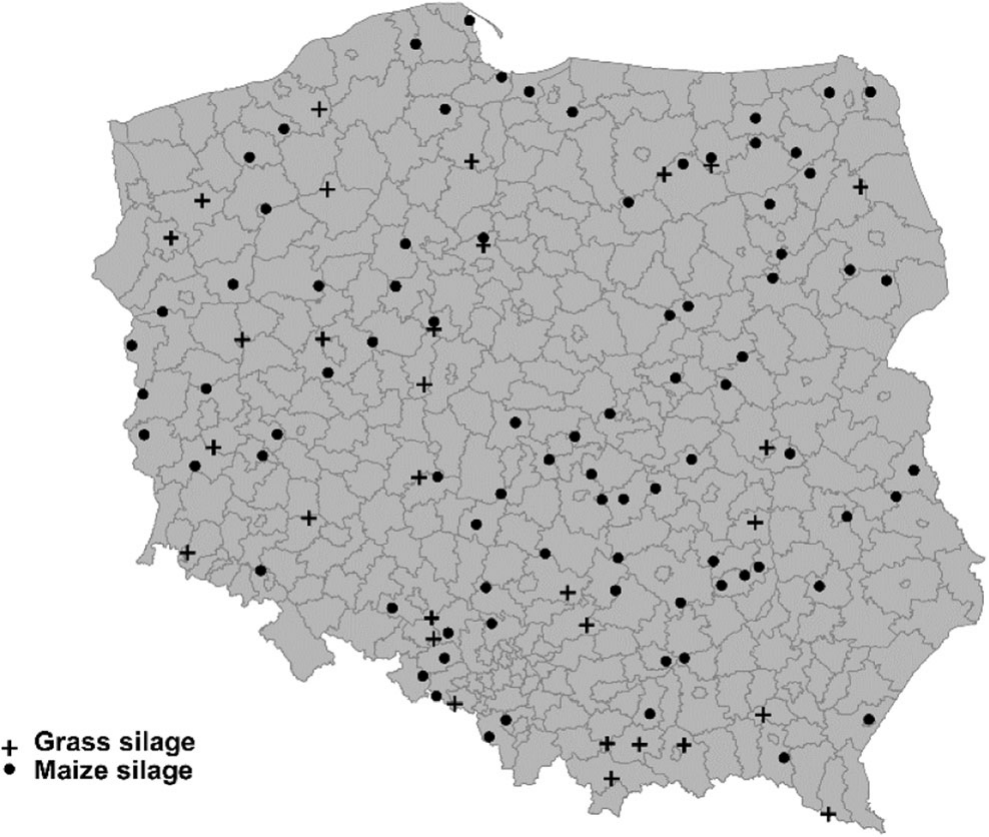
Map of Poland illustrating localization of surveyed samples

One hundred twenty visibly mould-free samples of silage, consisting of maize (87) and grass (33), were collected from 16 provinces (voivodeships) of Poland, with eight samples coming from each region (Fig. 1). Samples were collected between July and December of 2015 by the Veterinary Inspection officers working with feed manufacturers. The types of silage sampled were representative of the different regions of Poland, and were taken in compliance with European regulations (European Commission 2009), as part of a national monitoring programme. The samples, weighing about 5 kg each, were divided separately into 1-kg subsamples, frozen and chopped (grass). Silage was homogenised (using a Waring Blender 8010EB, USA), and stored in the dark at − 20 °C until the date of analysis.

### Sample preparation

The protocol for sample preparation was adapted from a previous study (Jedziniak et al. 2016), with some modifications. Five grams of sample was placed into a glass tube and extracted using a 20 ml of mixture consisting of acetonitrile:water:formic acid (79:20:1, *v*/*v*/*v*), with a homogeniser (Polytron PT 3000, Switzerland) for 2 min (2240 × g). The sample was then put into storage for 12 h at 4 °C. Subsequently, the whole sample was put into a 50-ml polypropylene tube and shaken vertically (200 cycles/min) for 30 min. The sample was centrifuged (2643 × g, 15 min), and 2 ml of supernatant was transferred to a plastic test tube containing MgSO_4_ (150 mg) and C18 bulk sorbent (50 mg). The aliquot was immediately shaken vigorously for 30 s then centrifuged (2643 × g, 15 min). The extract (1 ml) was evaporated (40 °C) to dryness in a gentle stream of nitrogen. The dry residue was reconstituted with 500 μl of mobile phase A and 500 μl of mobile phase B (see the “LC-MS/MS conditions” section), and then sample was transferred to a 1.5-ml polypropylene tube for centrifuging for 30 min (16,602 × g). The extract was put into an autosampler vial and 5 μl was injected for UPLC-MS/MS analysis. For each analysis series, matrix-matched calibration curves were prepared at three levels (0.5 × GL, 1 × GL, 2 × GL), for both the maize and grass silage.

### LC-MS/MS conditions

Chromatographic separation was performed for 16 min on a Kinetex BiPhenyl column (100 × 2.1 mm; particle size 2.6 μm), coupled with a BiPhenyl security guard cartridge (Phenomenex, Torrance, CA, US). The column oven temperature was set to 40 °C. The gradient elution had flowrate of 0.3 ml/min. Mobile phase A consisted of 10 mM ammonium acetate and 0.1% acetic acid/MeOH (95:5, *v*/*v*). Mobile phase B consisted of 10 mM ammonium acetate and 0.1% acetic acid/MeOH (5:95, *v*/*v*), was used as follows: (1) linear gradient to 95% solvent B 0–9 min; (2) 95% solvent B held from 9 to 13 min and (3) column reconditioning with the initial composition of the mobile phase at 13–15.9 min.

The analyses were performed with a Nexera X2 system, coupled with a LCMS-8050 triple quadrupole mass spectrometer (Shimadzu, Japan), equipped with an electrospray and operated in positive (ESI +) and negative (ESI −) modes using fast polarity switching, controlled by LabSolution 5.60 SP2 software. Two multiple reaction monitoring (MRM) transitions for each analyte were monitored (Supplementary Table S1). The time of analysis was divided by time segments (retention time ± 2 min), each acquiring different MRM transitions. The following parameters were used: resolution Q1 and Q3 unit; nebulising gas flow, 2 L/min; heating gas flow, 10 L/min; drying gas flow, 10 L/min; interface temperature, 300 °C; desolvation line temperature, 250 °C and heat block temperature, 400 °C.

### Method validation

During the validation process, the following parameters were determined for the maize and grass silage: linearity range; limit of detection (LOD, μg/kg); limit of quantification (LOQ, μg/kg); recovery (REC, %) and repeatability expressed as relative standard deviation (RSD, %). LOD and LOQ were calculated based on a signal-to-noise (S/N) ratio of peaks (LOD, S/N = 3; LOQ, S/N = 10). The results were checked by analysis of the background noise of 20 different pseudo-blank silage samples (Schaechtele and Robouch 2016). Repeatability was determined using pseudo-blank samples of maize and grass silage spiked at 1 GL (Supplementary Table S2), with a working solution in six repetitions over two different days. For the recoveries study, the same samples were used and compared with concentration of standard solution. For the linearity range, five-point matrix-match calibration curves were prepared by spiking pseudo-blank samples at different levels (0.25 GL, 0.5 GL, 1 GL, 2 GL, 5 GL). Additionally, according to Matuszewski et al. (2003), the matrix effect (ME) for each mycotoxins was evaluated as a ratio of the concentration of pre-spiked and post-spiked samples (in three repetitions) at 1 GL.

### Statistical analysis

The correlations between mycotoxin concentrations were calculated using STATISTICA, version 10 (StatSoft, Inc. 2011), with a Spearman correlation test used for this purpose. The correlation was considered significant at a level of *p* = 0.05. To assess the significance of the differences in the results between concentrations of mycotoxins in maize and grass silage, a Mann-Whitney rank sum test was performed (*p* value of < 0.05 was regarded as significant).

## Results and discussion

### Method validation

The LC-MS/MS method was successfully validated for all analytes for maize and grass silage (Supplementary Table S3). The obtained results demonstrated sufficient linearity, with *R*^2^ above 0.98 for most of the analytes in both matrixes. Calculated recoveries ranged between 70 and 120% for 19 and 13 out of compounds for maize and grass silage, respectively. The RSD measured during the repeatability study did not exceed 30%. LODs and LOQs ranged from 0.06 to 15.0 μg/kg and 0.20 to 50.0 μg/kg, respectively. The lowest LOQ was obtained for ENNs, STC and BEA, both for maize and grass silage. In the field of ubiquitous contaminants, blank samples may not always be available. As an alternative, low contaminated samples (pseudo-blanks) were used in this study. The evaluation of ME for both matrixes demonstrated large variation between analytes (Supplementary Fig. S1). In the case of maize silage, 75% of the compounds fell into a range of 70–120%, and in turn, in the grass silage, only 45% of the analytes were in this range. For example, DON in maize showed a slight enhancement effect (109%), while grass had a matrix suppression effect (62%). The observed results (recovery and ME) indicated the need to quantitate mycotoxins in various commodities, by preparing matrix-matched calibration curves for both matrixes, in each analysis. This has also been pointed out by other researchers (Rasmussen et al. 2010; Dzuman et al. 2014; Dagnac et al. 2016).

### Frequency and levels of mycotoxins in the silage

The relatively high frequency of mycotoxins as obtained in our study was because of the low LOQs obtained by the LC-MS/MS method used for analysis. In general, the results are generally in line with other surveys’ results in our region (Grajewski et al. 2012; Zachariasova et al. 2014).

**Fig. 2:**
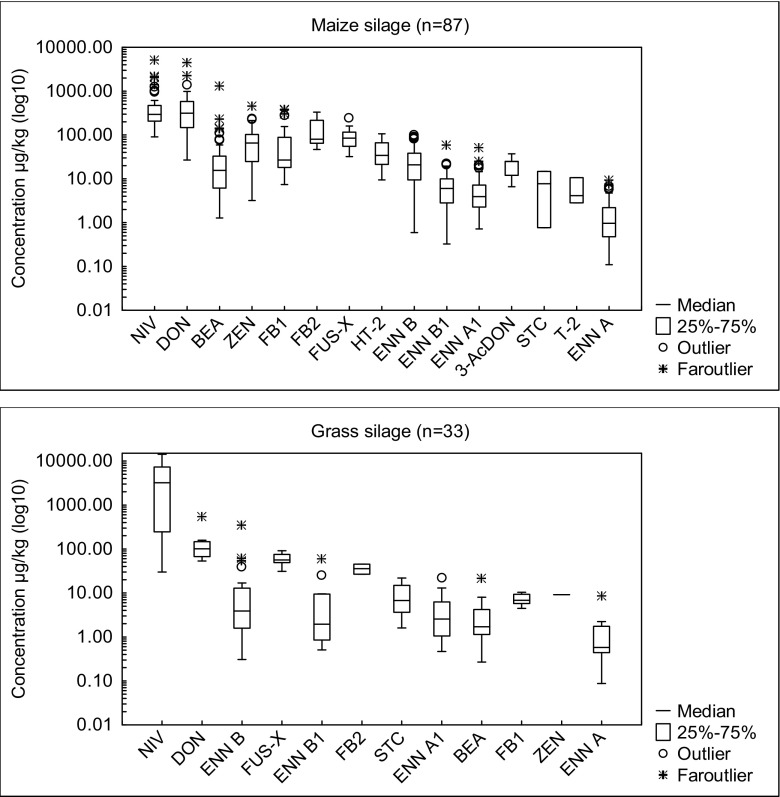
Box plot presentation of mycotoxins levels in maize and grass silage

Overall, the data revealed the presence of 15 and 12 different analytes in maize and grass silage, respectively. BEA, DON, HT-2, ENNs (ENN A, ENN A_1,_ ENN B, ENN B_1_,) NIV and ZEN (Fig. 2) were frequently found in all samples. Detailed information on the concentrations and prevalence of the detected toxins is compiled in Supplementary Table S4. With respect to maize by-products, all of the regulated mycotoxins were below the EU guidance values (European Commission 2006). The *Fusarium* toxins DON and ZEN were amongst the most frequently encountered mycotoxins in maize silage and were found in 82% and 57%, of the samples, with average concentrations at levels of 447 μg/kg (DON) and 82.4 μg/kg (ZEN). Nearly half of the positive samples contained less than 200 μg/kg and 100 μg/kg, respectively (Supplementary Fig. S2). Similar results were obtained by Kosicki et al. (2016) who stated that DON and ZEN were the most frequent toxins, and detected in 86% and 88% of positive-maize samples, respectively. In contrast, the results of Storm et al. (2014) are contrary to our data, as the authors determined DON in only 6% of examined samples, compared to the 82% reported in our study.

**Fig. 3:**
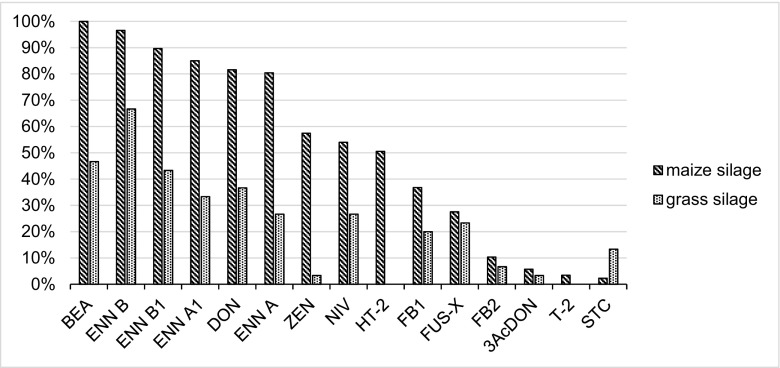
Frequency of mycotoxins in maize and grass silage

Maize and grass silage showed qualitative and quantitative differences with regard to mycotoxin contamination. Except for STC, all compounds were more often found in the maize silage than in grass silage (Fig. 3). This is probably a result of the fact that fungi and other pathogens can easily survive on the maize crops, which are richer in necessary proteins and polysaccharides than the grass (Zachariasova et al. 2014). The differences between concentrations of mycotoxins in these two types of silage were statistically significant (*p* < 0.05) for BEA, DON, ENNs, FB1, HT-2, NIV, STC and ZEN. In our study, DON and ZEN were detected in grass silage at a frequency of 37% and 3%, respectively. In the literature, information on the occurrence of mycotoxins in grass silage is scarce, especially for *Fusarium* toxins. In the studies conducted by Skladanka et al. (2013), the content of several mycotoxins in grass silage was assessed, with maximum content of DON at 167 μg/kg and ZEN at 66.9 μg/kg. These findings support our observation that DON could be present in grass silage and is probably produced by *Fusarium* species during their growth in the field. Moreover, Cavallarin et al. (2004) suggest that *Fusarium* mycotoxins could be produced within silage, as they detected ZEN in high concentration in grass silage (above 300 μg/kg), whereas Driehuis et al. (2008) detected ZEN in 6% of surveyed samples of grass silage. The authors verified that the occurrence of toxins in maize silage is higher than in grass or wheat silage. Low level of ZEN was also quantified in grass silage in the study (McElhinney et al. 2016) (mean 53 μg/kg) and was the only EU-regulated mycotoxin detected in surveyed samples. ZEN was found in the 43% of unfermented hay in the German study (Schollenberger et al. 2006).

FUS-X and NIV are 8-ketotrichothecenes structurally related to DON. The mean level of NIV was significantly higher in grass than in maize silage, namely 4473 μg/kg versus 544 μg/kg, respectively. In four samples, the concentration of NIV exceeded a value of 5000 μg/kg; however, these samples constituted only a small percentage of silage (3.33% of positives). Several authors have previously reported the occurrence of NIV in maize silage (Oldenburg and EIIner 2005; Schollenberger et al. 2006; Storm et al. 2014). The average level in these studies ranged from 263 to even 1612 μg/kg. In hay, Schollenberger et al. (2006) reported a frequency of 4% with an average NIV concentration level of 131 μg/kg. In our survey, FUS-X was detected both in maize and grass silage at similar average concentration 92 and 59 μg/kg, respectively. Lastly, Zachariasova et al. (2014) noted that the mean concentration of FUS-X in maize silage was 77 μg/kg, while in grass was not determined.

The T-2 and HT-2 toxins (type A trichothecenes) were observed only in maize silage, with a frequency of 3% and 51% in mycotoxin-positive samples, respectively. Moreover, concentrations of T-2 toxin constituted only 10% of HT-2 toxin’s average content (5.90 and 43.2 μg/kg, respectively). Generally, our results are in line with Grajewski et al. (2012), especially in cases of high content of HT-2 toxin (45.6 μg/kg) compared to a lower T-2 toxin content (3.10 μg/kg). This could be explained by the fact that T-2 toxin is rapidly metabolised to HT-2 toxin during the fermentation process in silage.

Regarding AFs and OTA, these toxins were not present in any sample, similarly to the findings of Driehuis et al. (2008) and Zachariasova et al. (2014). AFs and OTA are produced by storage fungi (*Aspergillus* or *Penicillium* fungi) under favourable storage conditions, such as excessive humidity. Ensiling maize limits the available oxygen and water content, which probably prevents storage moulds from growing. However, in Europe, a few authors have reported the occurrence of AFB_1_ in maize silage (Garon et al. 2006; Tsiplakou et al. 2014). The differences in occurrence of the main mycotoxins detected in central and eastern Europe (DON, NIV and ZEN) could be caused by a climatic differences. The warmer, more humid Mediterranean climate creates favourable conditions for the growth of moulds producing, for example, AFB_1_.

Of the so-called emerging mycotoxins, BEA was the most commonly detected, with a presence in 108 samples (87%) and average and maximum concentrations of 35.8 μg/kg and 1309 μg/kg, respectively. Nevertheless, the mean content of this toxin in positive samples was low—less than 50 μg/kg in 85% of the samples contaminated with BEA (Supplementary Fig. S2). Finally, ENN A, ENN A_1_, ENN B and ENN B_1_ were the most prevalent toxins in the investigated silage, with a presence in 66%, 71%, 89% and 78% of tested samples, respectively. ENNs were two times often detected in maize than grass silage (88% and 43%, respectively). The highest concentrations were found for ENN B (344 μg/kg). However, in many cases, ENNs were found in low concentrations, mostly less than 10 μg/kg (Supplementary Fig. S2). Our results for BEA and the four ENNs are similar as reported by other authors (Dagnac et al. 2016), who reported that the most frequently detected toxin was ENN B (51%), in an average concentration of 393 μg/kg. The high prevalence of BEA and ENNs has already been described by McElhinney et al. (2016), who state that these toxins to be the most prevalent in pit and bale silage (less than 50% and 60% of samples, respectively). Still, no information is currently available on the adverse effects of ENNs and BEA on animal health, or their possible combined effects (EFSA 2014a).

### Co-occurrence of the detected mycotoxins

Statistical analysis confirmed our finding that samples with a relatively high concentration of DON (699 μg/kg) were often contaminated with FUS-X (113 μg/kg), NIV (615 μg/kg) or ZEN (444 μg/kg) (Table 1). Moreover, in few cases, DON was quantified simultaneously with its analogue—3-AcDON; however, the ratio 3-AcDON/DON was below 4%. Co-contamination of maize with DON and it analogues (3-AcDON, 15-AcDON and NIV) was already reported (Oldenburg and EIIner 2005). Our results are similar as that reported by Eckard et al. 2011, who rarely found 3-AcDON and only in samples with high total *Fusarium* toxins concentration.

**Table 1:** Co-contamination of selected silage samples having the highest overall toxin concentration

	NIV [μg/kg]	DON [μg/kg]	BEA [μg/kg]	ZEN [μg/kg]	FB1 [μg/kg]	ENA B [μg/kg]	FB2 [μg/kg]	FUS-X [μg/kg]	HT-2 [μg/kg]	ENA B1 [μg/kg]	ENA A1 [μg/kg]	3-AcDON [μg/kg]	STC [μg/kg]	T2 [μg/kg]	ENA A [μg/kg]
Sample 01*	1238	553	34.6	50.2	–	15.2	–	–	–	9.07	8.92	–	14.8	–	9.14
Sample 02*	1989	596	140	63.8	–	53.6	–	–	49.5	19.5	25.1	–	–	–	4.73
Sample 03*	1034	369	12.9	–	–	16.5	–	–	9.41	3.35	1.54	–	–	–	–
Sample 04**	14,262	158	20.8	–	–	4.64	–	–	–	5.47	6.24	–	22.0	–	8.57
Sample 05*	2165	1404	35.3	64.8	91.9	67.9	65.0	–	16.2	16.2	9.03	25.1	–	–	0.54
Sample 06*	1413	4347	110	228	42.4	66.3	–	–	78.1	14.2	11.0	–	–	–	2.58
Sample 07*	375	503	21.8	235	–	72.1	–	–	12.8	10.4	6.64	–	–	–	1.10
Sample 08*	474	983	7.78	75.8	–	29.6	–	–	–	6.24	3.83	–	–	–	0.34
Sample 09*	468	887	16.9	115	8.79	28.0	–	79.8	68.9	7.43	5.42	–	–	–	1.30
Sample 10*	135	749	12.0	214	9.84	38.1	–	–	22.3	3.25	1.10	–	–	–	–
Sample 11*	233	699	79.3	97.4	367	89.4	333	38.8	91.0	13.7	8.11	24.9	–	10.7	3.46
Sample 12*	266	224	31.3	20.7	299	101	267	32.3	27.3	57.2	51.2	–	–	–	6.68
Sample 13*	955	2210	228	173	88.4	82.7	46.8	95.1	87.3	21.7	17.7	37.2	–	2.85	5.72
Sample 14*	217	197	59.2	19.6	26.9	17.6	–	–	24.6	7.88	9.44	–	–	–	2.77
Sample 15*	228	939	33.6	175	18.1	82.7	–	100	107	10.7	4.09	–	–	–	0.82
Sample 16*	615	699	41.7	444	–	36.3	–	113		3.85	1.69	–	–	–	0.52
Sample 17*	316	354	6.46	24.9	–	24.5	–	–	60.3	4.90	2.54	–	–	–	0.67
Sample 18*	261	235	30.4	103	–	41.3	–	–	79.2	9.49	4.02	–	–	–	0.68

*maize silage, **grass silage

The outcome described above clearly demonstrates that the occurrence of fungi’s secondary metabolites in silage is relatively high, and that some samples were co-contaminated with several toxins, although at low concentration. All of the samples contained at least one mycotoxin, 61% of the samples were contaminated with at least five toxins (Supplementary Fig. S3). Regarding the co-occurrence of major *Fusarium* mycotoxins (BEA, DON, ENNs, HT-2, NIV and ZEN), 24 of our 120 samples contained all of these compounds (Supplementary Fig. S4). The observation is in agreement with the studies of Zachariasova et al. (2014), who noted co-contamination of multiple mycotoxins in maize silage with DON, ENNs, BEA and ZEN. Those authors reported each tested sample to be positive for at least one mycotoxin at a quantifiable level, with the simultaneous presence of DON and ENNs in the positive-surveyed samples. In our study, a significant number of silage samples (42%) both DON and ZEN were present simultaneously and a high correlation of co-occurrence between these toxins in maize silage was also noted (*r* = 0.74, *p* < 0.05) (Supplementary Table S5). The positive correlation between DON and ZEN in maize silage was previously observed by Kosicki et al. (2016). Our findings on the co-occurrence between of DON and ZEN are partially in disagreement with the study conducted by Borutova et al. (2012), who observed a positive correlation in silage only between FB_2_ and ZEN, and between FB_1_ and FB_2_. This could probably be explained by the completely different weather conditions in their Asia-Oceania sample region, with preferable conditions for *Fusarium* species to produce FB_1_ and FB_2_, then DON. However, in a different matrix (raw maize samples), they found high positive correlation between DON and ZEN.

In the case of the four ENNs, they were simultaneously present in 61% of the positive samples (Supplementary Fig. S3) with high positive correlation between ENN B and ENN B_1_ (*r* = 0.90, *p* < 0.05), the same pattern appearing in grass silage (*r* = 0.87, *p* < 0.05) (Supplementary Table S5).

The exposure to low concentrations of several mycotoxins may be of concern in terms of their potentially additive or synergistic effects on animals. In the study conducted by Alassane-Kpembi et al. (2013), the interactions between B-type trichotecenes (DON, 3-AcDON, 15-AcDON, FUS-X and NIV) on intestinal epithelial cells were assessed. The authors reported that the combination of toxins had an additive effect. These results demonstrate that the simultaneous presence of mycotoxins, in this case, for example, DON and NIV, can be more toxic than the toxicity predicted for one mycotoxin itself. The presence of multiple mycotoxins in animal feed could be considered as a potential source of health problems; however, co-contamination of samples at levels describes in our study seems to be less important than contamination with one toxin at higher concentration (e.g. NIV at maximum level 14,262 μg/kg).

The dimensions of the potential problem related to the co-occurrence of multiple mycotoxins in silage still have not been fully evaluated, especially in the case of emerging mycotoxins. Silage produced in Poland during the period of this survey was frequently contaminated with DON and ZEN, albeit at relatively low levels. It has to be emphasised that concentrations of all regulated toxins were considerably lower than the guideline values recommended by the European Commission. In some cases, concentration of emerging mycotoxins (BEA, NIV) was at possibly relevant levels. Moreover, the co-occurrence of the toxins was high, and the impact of their mixture could pose chronic problems for exposed cattle, with possible synergistic and/or additive effects. Higher frequency and concentrations for almost toxins were in maize than in grass silage. Putting these results in the context of mycotoxins exposure to animals health’s suggests that grass silage could be a “safer” option as source of animal feed. Therefore, multi-toxin monitoring should be increased in order to provide the information on the occurrence of different classes of mycotoxins simultaneously in different feed commodities. Further data on the toxicity of mixtures of mycotoxins are needed, in order to establish safe limits specifically for silage.

## Electronic supplementary material

ESM 1 (DOCX 387 kb)
