# Supplementary material for: Frequency and levels of regulated and emerging mycotoxins in silage in Poland
Source: Mycotoxin Res. 2018 Aug 22;35(1):17–25. doi: 10.1007/s12550-018-0327-0 (PMC6331501; doi:10.1007/s12550-018-0327-0)
Supplement: Supplementary file 1 — (DOCX 387 kb) [file 12550_2018_327_MOESM1_ESM.docx]

**Supplementary information**

**Supplementary figure S1.** Comparison of matrix effect for all validated analytes in maize and grass silages

FUS-X fusarenon X, 3-AcDON 3-acetyldeoxynivalenol, 15-AcDON 15-acetyldeoxynivalenol, FB_1_ fumonisin B_1_, HT-2 HT-2 toxin, DAS diacetoxyscirpenol, FB_2_ fumonisin B_2_, T-2 T-2 Toxin, OTA ochratoxin A, AFG_2_ aflatoxin G_2_, AFG_1_ aflatoxin G_1_, AFB_2_ aflatoxin B_2_, AFB_1_ aflatoxin B_1_, ENN B enniatin B, ENN B_1_ enniatin B_1_, STG sterigmatocystin, ENN A_1_ enniatin A_1_, ENN A enniatin A, BEA beauvericin, NIV nivalenol, DON deoxynivalenol, CIT citrinin, β-ZEL β-zearalenol, ZEN zearalenone

**Supplementary figure S2.** Distribution of most prevalent mycotoxins concentration range (maize and grass silages)

DON deoxynivalenol, ZEN zearalenone, ENNs enniatins, ENN B enniatin B, ENN B_1_ enniatin B_1_, ENN A_1_ enniatin A_1_, ENN A enniatin A, BEA beauvericin

**Supplementary figure S3.** Percentage of metabolites co-occurring in all of positive silage samples

**Supplementary figure S4.** Co-occurrence group of the most common prevalent mycotoxins in positive samples

NIV nivalenol, DON deoxynivalenol, ZEN zearalenone, HT-2 HT-2 toxin, ENNs enniatins, BEA beauvericin

**Supplementary table S1.** Optimized LC-MS/MS conditions

| Ionisation | Analyte | **Precursor ion (*m/z)*** | **Product ions (*m/z*)** | **Retention time (min)** |
| --- | --- | --- | --- | --- |
| **ESI ^+^** | FUS-X | [M+H]^+^ 355 | 337^a^, 229, 175 | 5.25 |
|  | 3-AcDON | [M+H]^+^ 339 | 231^a^, 203, 279 | 6.80 |
|  | 15-AcDON | [M+H]^+^ 339 | 256^a^, 279, 261 | 6.80 |
|  | FB_1_ | [M+H]^+^ 722 | 352^a^, 334 | 8.0 |
|  | HT-2 | [M+Na]+ 447 | 345^a^, 285 | 8.5 |
|  |  | [M+NH4]+ 442 | 263^a^, 215 | 8.5 |
|  | DAS | [M+NH_4_]^+^ 384 | 307^a^, 247, 247 | 8.7 |
|  | FB_2_ | [M+H]^+^ 706 | 336^a^, 318 | 9.0 |
|  | T-2 | [M+Na]^+^ 489 | 327^a^, 387 | 9.2 |
|  | OTA | [M+H]^+^ 404 | 239^a^, 358 | 9.5 |
|  | AFG2 | [M+H]+ 331 | 313^a^, 245, 189 | 10.0 |
|  | AFG1 | [M+H]+ 328 | 243^a^, 311 ,200 | 10.5 |
|  | AFB2 | [M+H]+ 315 | 287^a^,259,243 | 10.7 |
|  | AFB1 | [M+H]+ 313 | 285^a^, 241, 269 | 11.0 |
|  | ENN B | [M+H]^+^ 657 | 640^a^, 196, 86 | 11.5 |
|  | ENN B_1_ | [M+H]^+^ 671 | 654^a^, 196, 210 | 11.6 |
|  | STC | [M+H]^+^ 325 | 281^a^, 310, 253 | 11.7 |
|  | ENN A_1_ | [M+H]^+^ 685 | 668^a^, 210, 100 | 11.8 |
|  | ENN A | [M+H]^+^ 699 | 682^a^, 210, 100 | 12.0 |
|  | BEA | [M+NH_4_]^+^ 801 | 244, 134^a^, 784 | 12.6 |
| **ESI^-^** | NIV | [M-CH_3_HCOO]^-^ 371 | 311^a^, 281, 59 | 4.20 |
|  | DON | [M-CH_3_HCOO]^-^ 355 | 295, 265^a^, 59 | 4.50 |
|  | CIT | [M-H]^-^ 281 | 249^a^, 205 | 7.70 |
|  | β-ZEL | [M-H]- 319 | 275^a^, 130 | 9.00 |
|  | ZEN | [M-H]^-^ 317 | 175^a^, 131 | 9.90 |

^a^ - ion used for quantitation

FUS-X fusarenon X, 3-AcDON 3-acetyldeoxynivalenol, 15-AcDON 15-acetyldeoxynivalenol, FB_1_ fumonisin B_1_, HT-2 HT-2 toxin, DAS diacetoxyscirpenol, FB_2_ fumonisin B_2_, T-2 T-2 Toxin, OTA ochratoxin A, AFG_2_ aflatoxin G_2_, AFG_1_ aflatoxin G_1_, AFB_2_ aflatoxin B_2_, AFB_1_ aflatoxin B_1_, ENN B enniatin B, ENN B_1_ enniatin B_1_, STC sterigmatocystin, ENN A_1_ enniatin A_1_, ENN A enniatin A, BEA beauvericin, NIV nivalenol, DON deoxynivalenol, CIT citrinin, β-ZEL β-zearalenol, ZEN zearalenone

**Supplementary table S2.** Working solution containing 24 analytes in concentration, which was used to fortified samples at level corresponding to the lowest guidance levels (GL) relative to feeding stuff (European Commission, 2006, 2013) as well as concentration each toxins in this solution

|  | 1GL (µg/kg) | Concentration in working  solution (µg/ml) |
| --- | --- | --- |
| FUS-X | 100 | 1.10 |
| 3-AcDON | 100 | 1.10 |
| 15-AcDON | 100 | 1.10 |
| FB_1_ | 450 | 5.00 |
| HT-2 | 50 | 0.555 |
| DAS | 100 | 1.10 |
| FB_2_ | 450 | 5.00 |
| T-2 | 50 | 0.555 |
| OTA | 50 | 0.555 |
| AFG2 | 5 | 0.0555 |
| AFG1 | 5 | 0.0555 |
| AFB2 | 5 | 0.0555 |
| AFB1 | 5 | 0.0555 |
| ENN B | 100 | 1.10 |
| ENN B_1_ | 100 | 1.10 |
| STC | 100 | 1.10 |
| ENN A_1_ | 100 | 1.10 |
| ENN A | 100 | 1.10 |
| BEA | 100 | 1.10 |
| NIV | 900 | 10.0 |
| DON | 900 | 10.0 |
| CIT | 100 | 1.10 |
| β-ZEL | 100 | 1.10 |
| ZEN | 100 | 1.10 |

GL guidance level,

FUS-X fusarenon X, 3-AcDON 3-acetyldeoxynivalenol, 15-AcDON 15-acetyldeoxynivalenol, FB_1_ fumonisin B_1_, HT-2 HT-2 toxin, DAS diacetoxyscirpenol, FB_2_ fumonisin B_2_, T-2 T-2 Toxin, OTA ochratoxin A, AFG_2_ aflatoxin G_2_, AFG_1_ aflatoxin G_1_, AFB_2_ aflatoxin B_2_, AFB_1_ aflatoxin B_1_, ENN B enniatin B, ENN B_1_ enniatin B_1_, STC sterigmatocystin, ENN A_1_ enniatin A_1_, ENN A enniatin A, BEA beauvericin, NIV nivalenol, DON deoxynivalenol, CIT citrinin, β-ZEL β-zearalenol, ZEN zearalenone

**Supplementary table S3.** Validation results of mycotoxins determination by LC-MS/MS in maize and grass silage

|  | maize silage | | | | | | grass silage | | | | | |
| --- | --- | --- | --- | --- | --- | --- | --- | --- | --- | --- | --- | --- |
|  | **Recovery [%] ±SD** | **Repeatability [%]** | **LOD**  **[µg/kg]** | **LOQ**  **[µg/kg]** | **linearity range [µg/kg]** | **R^2^** | **Recovery [%] ±SD** | **Repeatability**  **[%]** | **LOD**  **[µg/kg]** | **LOQ**  **[µg/kg]** | **linearity range [µg/kg]** | **R^2^** |
| FUS-X | 123 ± 18 | 14 | 7.50 | 25.0 | 25.0-500 | 0.970 | 98 ± 21 | 21 | 9.00 | 30.0 | 30.0-500 | 0.998 |
| 3-ADON | 89 ± 10 | 7 | 1.80 | 6.00 | 6.00-500 | 0.993 | 94 ± 7 | 17 | 3.00 | 10.0 | 10.0-500 | 0.999 |
| 15-ADON | 97 ± 8 | 17 | 15.0 | 50.0 | 50.0-500 | 0.994 | 82 ± 9 | 12 | 15.0 | 50.0 | 50.0-500 | 0.994 |
| FB_1_ | 97 ± 7 | 4 | 0.90 | 3.00 | 3.00-2250 | 0.997 | 86 ± 4 | 3 | 1.20 | 4.00 | 4.00-2250 | 0.940 |
| HT-2 | 46 ± 5 | 3 | 0.90 | 3.00 | 3.00-500 | 0.990 | 17 ± 2 | 11 | 7.50 | 25.0 | 25.0-500 | 0.996 |
| DAS | 87 ± 3 | 4 | 0.90 | 3.00 | 3.00-500 | 0.998 | 71 ± 3 | 3 | 1.50 | 5.00 | 5.00-500 | 0.999 |
| FB_2_ | 102 ± 8 | 5 | 2.10 | 7.00 | 7.00-2250 | 0.990 | 102 ± 10 | 4 | 6.00 | 20.0 | 20.0-2250 | 0.980 |
| T-2 | 111 ± 6 | 3 | 0.90 | 3.00 | 3.00-500 | 0.999 | 89 ± 3 | 4 | 4.50 | 15.0 | 15.0-500 | 0.976 |
| OTA | 87 ± 3 | 4 | 0.54 | 1.80 | 1.80-500 | 0.990 | 75 ± 1 | 2 | 0.60 | 2.00 | 2.00-500 | 0.980 |
| AFG_2_ | 88 ± 8 | 24 | 0.38 | 1.25 | 1.25-25 | 0.980 | 159 ± 7 | 30 | 1.50 | 5.00 | 5.00-25.0 | 0.994 |
| AFG_1_ | 71 ± 7 | 12 | 0.38 | 1.25 | 1.25-25 | 0.940 | 87 ± 12 | 8 | 1.50 | 5.00 | 5.00-25.0 | 0.975 |
| AFB_2_ | 82 ± 6 | 25 | 0.38 | 1.25 | 1.25-25 | 0.992 | 87 ± 2 | 9 | 1.50 | 5.00 | 5.00-25.0 | 0.992 |
| AFB_1_ | 71 ± 11 | 7 | 0.38 | 1.25 | 1.25-25 | 0.998 | 69 ± 4 | 6 | 0.75 | 2.50 | 2.50-5.00 | 0.980 |
| ENN B | 75 ± 2 | 4 | 0.06 | 0.20 | 0.20-500 | 0.994 | 56 ± 3 | 3 | 0.15 | 0.50 | 0.50-500 | 0.996 |
| ENN B_1_ | 71 ± 3 | 4 | 0.06 | 0.20 | 0.20-500 | 0.995 | 48 ± 2 | 2 | 0.15 | 0.50 | 0.50-500 | 0.981 |
| STC | 103 ± 14 | 2 | 0.24 | 1.25 | 1.25-500 | 0.999 | 81 ± 10 | 3 | 0.45 | 1.50 | 1.50-500 | 0.969 |
| ENN A_1_ | 74 ± 6 | 5 | 0.06 | 0.20 | 0.20-500 | 0.995 | 43 ± 6 | 4 | 0.15 | 0.50 | 0.50-500 | 0.965 |
| ENN A | 64 ± 3 | 5 | 0.06 | 0.20 | 0.20-500 | 0.994 | 32 ± 2 | 2 | 0.15 | 0.50 | 0.50-500 | 0.992 |
| BEA | 54 ± 5 | 5 | 0.06 | 0.20 | 0.20-500 | 0.995 | 19 ± 2 | 4 | 0.15 | 0.50 | 0.50-500 | 0.991 |
| NIV | 97 ± 10 | 4 | 9.00 | 30.0 | 30.0-4500 | 0.990 | 58 ± 4 | 5 | 9.00 | 30.0 | 30.0-4500 | 0.994 |
| DON | 91 ± 6 | 4 | 9.00 | 30.0 | 30.0-4500 | 0.987 | 61 ± 3 | 4 | 15.0 | 50.0 | 50.0-4500 | 0.997 |
| CIT | 148 ± 19 | 9 | 2.40 | 8.00 | 8.00-500 | 0.990 | 70 ± 8 | 4 | 2.40 | 8.00 | 8.00-500 | 0.970 |
| β-ZEL | 76 ± 6 | 11 | 3.90 | 13.0 | 13.0-500 | 0.999 | 36 ± 5 | 8 | 4.50 | 15.0 | 15.0-500 | 0.960 |
| ZEN | 108 ± 10 | 3 | 0.45 | 1.50 | 1.50-500 | 0.999 | 92 ± 6 | 3 | 0.90 | 3.00 | 3.00-500 | 0.978 |

SD standard deviation, Repeatability expressed as relative standard deviation at 1GL (guidance level), LOD limit of detection, LOQ limit of quantification
FUS-X fusarenon X, 3-AcDON 3-acetyldeoxynivalenol, 15-AcDON 15-acetyldeoxynivalenol, FB_1_ fumonisin B_1_, HT-2 HT-2 toxin, DAS diacetoxyscirpenol, FB_2_ fumonisin B_2_, T-2 T-2 Toxin, OTA ochratoxin A, AFG_2_ aflatoxin G_2_, AFG_1_ aflatoxin G_1_, AFB_2_ aflatoxin B_2_, AFB_1_ aflatoxin B_1_, ENN B enniatin B, ENN B_1_ enniatin B_1_, STC sterigmatocystin, ENN A_1_ enniatin A_1_, ENN A enniatin A, BEA beauvericin, NIV nivalenol, DON deoxynivalenol, CIT citrinin, β-ZEL β-zearalenol, ZEN zearalenone

**Supplementary table S4.** Mycotoxins concentration and occurrence detected in maize and grass silage

| Mycotoxins | NIV | DON | FUS-X | 3AcDON | FB_1_ | FB_2_ | HT-2 | T-2 | ZEN | STC | ENN B_1_ | ENN B | ENN A_1_ | ENN A | BEA |
| --- | --- | --- | --- | --- | --- | --- | --- | --- | --- | --- | --- | --- | --- | --- | --- |
| maize silage (87 samples) |  |  |  |  |  |  |  |  |  |  |  |  |  |  |  |
| mean (µg/kg) | 544 | 447 | 92.0 | 21.2 | 73.5 | 148 | 43.2 | 5.88 | 82.4 | 7.77 | 7.64 | 28.2 | 6.06 | 1.69 | 42.2 |
| median (µg/kg) | 295 | 314 | 84.9 | 24.9 | 26.5 | 80.3 | 34.1 | 4.12 | 65.8 | 7.77 | 6.03 | 20.9 | 3.93 | 0.97 | 15.6 |
| maximum (µg/kg) | 5105 | 4347 | 242 | 37.2 | 379 | 333 | 107 | 10.7 | 444 | 14.8 | 57.2 | 101 | 51.2 | 9.14 | 1309 |
| occurrence (%) | 54 | 82 | 28 | 6 | 37 | 10 | 51 | 3 | 57 | 2 | 90 | 97 | 85 | 80 | 100 |
| grass silage (33 samples) |  |  |  |  |  |  |  |  |  |  |  |  |  |  |  |
| mean (µg/kg) | 4473 | 139 | 59.1 | 12.3 | 7.24 | 35.8 | <LOD | <LOD | 9.14 | 9.27 | 9.18 | 28.3 | 5.35 | 1.77 | 3.76 |
| median (µg/kg) | 3208 | 101 | 56.2 | 12.3 | 6.80 | 35.8 | <LOD | <LOD | 9.14 | 6.75 | 1.94 | 3.89 | 2.55 | 0.57 | 1.71 |
| maximum (µg/kg) | 14262 | 528 | 91.6 | 12.3 | 10.4 | 45.0 | <LOD | <LOD | 9.14 | 22.0 | 58.0 | 345 | 21.6 | 8.57 | 20.8 |
| occurrence (%) | 27 | 37 | 23 | 3 | 20 | 7 | 0 | 0 | 3 | 13 | 43 | 67 | 33 | 27 | 47 |
| total (120) |  |  |  |  |  |  |  |  |  |  |  |  |  |  |  |
| mean (µg/kg) | 1116 | 406 | 84.5 | 19.7 | 63.0 | 128 | 43.2 | 5.88 | 80.8 | 8.77 | 7.70 | 27.5 | 5.92 | 1.69 | 35.8 |
| median (µg/kg) | 316 | 237 | 75.5 | 18.6 | 23.5 | 70.3 | 34.1 | 4.12 | 65.8 | 6.75 | 5.15 | 17.4 | 3.83 | 0.96 | 13.4 |
| maximum (µg/kg) | 14262 | 4347 | 242 | 37.2 | 379 | 333 | 107 | 10.7 | 444 | 22.0 | 58.0 | 345 | 51.2 | 9.14 | 1309 |
| occurrence (%) | 46 | 68 | 26 | 5 | 32 | 9 | 37 | 3 | 43 | 5 | 78 | 89 | 71 | 66 | 87 |

NIV nivalenol, DON deoxynivalenol, FUS-X fusarenon X, 3-AcDON 3-acetyldeoxynivalenol, FB_1_ fumonisin B_1_, FB_2_ fumonisin B_2_, HT-2 HT-2 toxin, T-2 T-2 Toxin, ZEN zearalenone, STC sterigmatocystin, ENN B_1_ enniatin B_1_, ENN B enniatin B, , ENN A_1_ enniatin A_1_, ENN A enniatin A, BEA beauvericin,

**Supplementary table S 5.** Correlation between individual mycotoxins in maize and grass silage

|  | Maize silage | | | | | | | | | | | | | |
| --- | --- | --- | --- | --- | --- | --- | --- | --- | --- | --- | --- | --- | --- | --- |
| Mycotoxin | **NIV** | **DON** | **FUS-X** | **3-AcDON** | **FB_1_** | **FB_2_** | **HT-2** | **T-2** | **ZEA** | **STC** | **ENN B_1_** | **ENN B** | **ENN A_1_** | **ENN A** |
| DON | 0.57^b^ |  |  |  |  |  |  |  |  |  |  |  |  |  |
| FUS-X | 0.04 | 0.07 |  |  |  |  |  |  |  |  |  |  |  |  |
| 3-AcDON | 0.14 | 0.22^a^ | 0.17 |  |  |  |  |  |  |  |  |  |  |  |
| FB_1_ | 0.14 | 0.17 | 0.47^a^ | 0.31 |  |  |  |  |  |  |  |  |  |  |
| FB_2_ | 0.10 | 0.11 | 0.25^a^ | 0.56^b^ | 0.62^b^ |  |  |  |  |  |  |  |  |  |
| HT-2 | 0.37^a^ | 0.54^b^ | 0.12 | 0.09 | 0.18 | 0.09 |  |  |  |  |  |  |  |  |
| T-2 | 0.12 | 0.21 | 0.14 | 0.77^b^ | 0.18 | 0.35^a^ | 0.15 |  |  |  |  |  |  |  |
| ZEA | 0.47^a^ | 0.74^b^ | 0.14 | 0.18 | 0.18 | 0.06 | 0.53^b^ | 0.18 |  |  |  |  |  |  |
| STC | 0.20^a^ | 0.19 | -0.09 | -0.04 | -0.11 | -0.05 | -0.06 | -0.03 | 0.16 |  |  |  |  |  |
| ENN B_1_ | 0.42^a^ | 0.54^b^ | 0.05 | 0.21^a^ | 0.17 | 0.23^a^ | 0.48^a^ | 0.16 | 0.53^b^ | 0.18 |  |  |  |  |
| ENN B | 0.44^a^ | 0.66^b^ | 0.02 | 0.25^a^ | 0.12 | 0.23^a^ | 0.56^b^ | 0.21^a^ | 0.66^b^ | 0.05 | 0.90^b^ |  |  |  |
| ENN A_1_ | 0.39^a^ | 0.34^a^ | 0.07 | 0.12 | 0.20^a^ | 0.24^a^ | 0.49^a^ | 0.07 | 0.41^a^ | 0.20^a^ | 0.84^b^ | 0.65^b^ |  |  |
| ENN A | 0.21^a^ | 0.18 | 0.03 | 0.05 | 0.07 | 0.18 | 0.50^b^ | 0.10 | 0.28^a^ | 0.17 | 0.57^b^ | 0.42^a^ | 0.85^b^ |  |
| BEA | 0.43^a^ | 0.44^a^ | 0.11 | 0.13 | 0.23^a^ | 0.27^a^ | 0.44^a^ | 0.10 | 0.43^a^ | 0.19 | 0.68^b^ | 0.57^b^ | 0.74^b^ | 0.60^b^ |
|  | **Grass silage** | | | | | | | | | | | | | |
| DON | 0.16 |  |  |  |  |  |  |  |  |  |  |  |  |  |
| FUS-X | 0.01 | 0.52^b^ |  |  |  |  |  |  |  |  |  |  |  |  |
| 3-AcDON | -0.10 | 0.27^a^ | 0.34^a^ |  |  |  |  |  |  |  |  |  |  |  |
| FB_1_ | -0.12 | 0.66^b^ | 0.68^b^ | 0.44^a^ |  |  |  |  |  |  |  |  |  |  |
| FB_2_ | 0.15 | 0.33_a_ | 0.17 | 0.67^b^ | 0.25^a^ |  |  |  |  |  |  |  |  |  |
| HT-2 | n.d. | n.d. | n.d. | n.d. | n.d. | n.d. |  |  |  |  |  |  |  |  |
| T-2 | n.d. | n.d. | n.d. | n.d. | n.d. | n.d. | n.d. |  |  |  |  |  |  |  |
| ZEA | 0.10 | 0.08 | 0.19 | -0.04 | -0.12 | -0.06 | n.d. | n.d. |  |  |  |  |  |  |
| STC | 0.55^b^ | 0.17 | -0.19 | -0.07 | -0.17 | -0.09 | n.d. | n.d. | -0.09 |  |  |  |  |  |
| ENN B_1_ | -0.02 | 0.04 | 0.05 | 0.20^a^ | -0.06 | 0.03 | n.d. | n.d. | 0.30^a^ | -0.09 |  |  |  |  |
| ENN B | -0.07 | -0.06 | -0.07 | 0.19 | -0.19 | 0.21^a^ | n.d. | n.d. | 0.35^a^ | -0.25 | 0.87^b^ |  |  |  |
| ENN A_1_ | 0.09 | 0.18 | 0.09 | -0.12 | -0.02 | -0.17 | n.d. | n.d. | 0.36^a^ | -0.01 | 0.71^b^ | 0.58^b^ |  |  |
| ENN A | 0.19 | 0.04 | 0.00 | -0.11 | -0.08 | -0.15 | n.d. | n.d. | 0.41^a^ | 0.26^a^ | 0.48^a^ | 0.33^a^ | 0.74^b^ |  |
| BEA | 0.36 | -0.08 | 0.00 | 0.18 | -0.10 | 0.00 | n.d. | n.d. | 0.27^a^ | 0.29^a^ | 0.54^b^ | 0.42^a^ | 0.43^a^ | 0.58^b^ |

Correlation is significant at the 0.05 level, ^a^ results with weak correlation 0.2<r<0.5, ^b^ results with high correlation r>0.5, n.d.-not detected

NIV nivalenol, DON deoxynivalenol, FUS-X fusarenon X, 3-AcDON 3-acetyldeoxynivalenol, FB_1_ fumonisin B_1_, FB_2_ fumonisin B_2_, HT-2 HT-2 toxin, T-2 T-2 Toxin, ZEA zearalenone, STC sterigmatocystin, ENN B_1_ enniatin B_1_, ENN B enniatin B, , ENN A_1_ enniatin A_1_, ENN A enniatin A, BEA beauvericin,
